# Heart failure in childhood cancer survivors—a systematic review protocol

**DOI:** 10.1186/s13643-022-01929-0

**Published:** 2022-03-29

**Authors:** Tove Berg, Jens Böhmer, Bright I. Nwaru, Kristjan Karason, Marianne Jarfelt

**Affiliations:** 1 Department of Pediatric Medicine, The Queen Silvia Children’s Hospital, Sahlgrenska University Hospital, Behandlingsvagen 7, 416 50 Gothenburg, Sweden; 2 Department of Pediatrics, Institute of Clinical Sciences, Sahlgrenska Academy, University of Gothenburg, Box 426, 405 30 Gothenburg, Sweden; 3 Pediatric Heart Center, The Queen Silvia Children’s Hospital, Sahlgrenska University Hospital, Behandlingsvagen 7, 416 50 Gothenburg, Sweden; 4 Krefting Research Centre, Institute of Medicine, University of Gothenburg, Box 424, 405 30 Gothenburg, Sweden; 5 Department of Cardiology, Sahlgrenska University Hospital, 413 45 Gothenburg, Sweden; 6 Transplant Institute, Sahlgrenska University Hospital, Bruna straket 5, 6th floor, 413 45 Gothenburg, Sweden; 7 Institute of Medicine, Sahlgrenska Academy, University of Gothenburg, Box 424, 405 30 Gothenburg, Sweden; 8 Long-term follow-up Clinic for Adult Childhood Cancer Survivors, Department of Oncology, Sahlgrenska University Hospital, Bla straket 2, 413 45 Gothenburg, Sweden; 9 Department of Oncology, Institute of Clinical Sciences, Sahlgrenska Academy, University of Gothenburg, Box 426, 405 30 Gothenburg, Sweden

**Keywords:** Childhood cancer, Anthracyclines, Survivors, Cardiomyopathy, Heart failure protocol and guidelines, Meta-analysis, Protocol and guidelines, Systematic review

## Abstract

**Background:**

Over the past decades, the survival rate for childhood cancer has greatly improved. However, the risk of late cardiac complications after cancer treatment remains high. Previous studies have shown that the risk for heart failure among childhood cancer survivors is significantly higher than that observed in varying control populations. The aim of this systematic review is to identify, critically appraise, and synthesize existing population-based studies reporting on the frequency of heart failure, both the incidence and prevalence, that may develop after treatment for childhood cancer.

**Method:**

The following databases will be searched from their inception date until May 17, 2021: MEDLINE, Embase, Scopus, CINAHL, CAB International, AMED, Global Health, PsycINFO, Web of Science, and Google Scholar. Population-based studies reporting on the incidence and/or prevalence of heart failure after the treatment of any type of childhood cancer will be included. The screening of articles, data extraction, and quality assessment will be performed independently by two reviewers. The quality and risk of bias in the included studies will be assessed by using the Effective Public Health Practice Project tool. A narrative synthesis of the extracted data will be carried out, and for studies that are sufficiently homogenous, a meta-analysis using random-effects models will be performed.

**Discussion:**

This systematic review will provide a clearer picture of the epidemiology of heart failure after the treatment of childhood cancer. The collected data will be of value for future childhood cancer treatment protocols and will offer guidance for posttreatment cardiac surveillance among survivors.

**Systematic review registration:**

PROSPERO CRD42021247622. Registered on April 28, 2021. This protocol follows the structure of the recommendation of the Preferred Reporting Items for Systematic Review and Meta-Analysis Protocols (PRISMA-P).

**Supplementary Information:**

The online version contains supplementary material available at 10.1186/s13643-022-01929-0.

## Background

The survival rate for childhood cancer has greatly improved over the past decades and is currently above 80%. As a result, the cohort of adult childhood cancer survivors is steadily growing [1–3]. Correspondingly, the risk of debilitating and sometimes fatal long-term side effects of cancer treatment is high, with a cumulative incidence of approximately 40% after 30 years of follow-up [4]. The most common forms of severe late complications and causes of death among childhood cancer survivors include secondary malignancies, cardiovascular diseases, and pulmonary disorders [5, 6]. Reports from the US Childhood Cancer Survivor Study have reported up to seven times higher risk of premature death due to cardiac complications among childhood cancer survivors compared to that among the general population [5, 6]. A wide variation in heart diseases in childhood cancer survivors has been reported [7–10]. The most common cardiac condition in this population is heart failure, which has previously been reported in a wide range, with up to a 15-fold higher risk compared to that of varying control populations [4, 7, 11, 12].

According to the 2013 American College of Cardiology Foundation (ACCF) and the American Heart Association (AHA) guidelines for the management of heart failure (HF), HF is largely a clinical diagnosis based on a careful history and physical examination and cannot be characterized by a single diagnostic test. The cardinal clinical manifestations of HF are dyspnea, fatigue, and fluid retention [13]. The severity of HF was initially defined by the New York Heart Association (NYHA) functional classification, in which patients are assigned to one of four groups based on how much they are limited during physical activity (Table 1) [14]. In addition, the ACCF/AHA has developed a classification including four stages that complements the NYHA system (Table 2) [13]. Both classifications provide useful information about the presence and severity of HF and have valuable prognostic implications. The ACCF and AHA have defined HF as a complex clinical syndrome that results from any structural or functional impairment of ventricular filling or the ejection of blood [13]. Accordingly, the European Society of Cardiology (ESC) states that HF is a clinical syndrome characterized by typical symptoms (e.g., breathlessness, ankle swelling, and fatigue) that may be accompanied by clinical signs (e.g., elevated jugular venous pressure, pulmonary crackles, and peripheral edema) caused by a structural and/or functional cardiac abnormality [15]

**Table 1:** The NYHA functional classification

Classification	Definition
I	No limitation of physical activity. Ordinary physical activity does not cause symptoms of HF.
II	Slight limitation of physical activity. Comfortable at rest, but ordinary physical activity results in symptoms of HF.
III	Marked limitation of physical activity. Comfortable at rest, but less than ordinary activity causes symptoms of HF.
IV	Unable to carry on any physical activity without symptoms of HF, or symptoms of HF at rest.

*NYHA* New York Heart Association, *HF* heart failure

**Table 2:** The ACCF/AHA stages of heart failure

Stage	Definition
A	At high risk for HF but without structural heart disease or symptoms of HF
B	Structural heart disease but without signs or symptoms of HF
C	Structural heart disease with prior or current symptoms of HF
D	Refractory HF requiring specialized interventions

*ACCF* American College of Cardiology Foundation, *AHA* American Heart Association, *HF* heart failure

Previous echocardiography investigations in childhood cancer survivors who did not experience any symptoms have reported a variety of structural and/or functional cardiac abnormalities, referred to as subclinical cardiotoxicity. Nevertheless, the observed frequency of such echocardiographic aberrations is highly inconsistent and ranges from 0 to 57% [16]. Furthermore, it is unclear to what degree subclinical cardiotoxicity in childhood cancer survivors may evolve into overt heart failure over time [16, 17].

Treatment with anthracyclines (ACs) has greatly improved survival rates in children with cancer [18]. A drawback is that ACs are cardiotoxic, and the risk for developing heart failure increases in parallel with the cumulative dose of these chemotherapeutics [8, 16, 19, 20]. A dose of anthracyclines below 250 mg/m^2^ has been reported to be associated with a low risk for cardiotoxicity, but for susceptible persons, no dose is safe [11, 20–22]. However, published data on which doses of different ACs are safe with respect to cardiotoxicity and the risk for subsequent heart failure are contradictory [23, 24]. In addition, other types of chemotherapeutic drugs have been seen to contribute to the cardiotoxic effect of ACs, including tyrosine kinase inhibitors, alkylating agents, and cisplatin [9]. Chest radiation is also a considerable risk factor for heart failure after treatment for childhood cancer, especially in combination with ACs, and may also contribute to coronary artery disease and valvular heart disease [8, 11, 25].

A synthesis of population-based studies reporting on the frequency of heart failure after childhood cancer treatment and examining whether the incidence and prevalence of this condition change over time is important. The acquisition of such epidemiological knowledge is likely to be of value when generating future treatment protocols for childhood cancer and organizing posttreatment cardiac surveillance programs.

## Objectives

The primary aim of this systematic review is to identify, critically appraise, and synthesize existing population-based studies reporting on the incidence and/or prevalence of heart failure in persons who have survived for a minimum of 5 years after treatment for childhood cancer. The incidence and prevalence of heart failure will be compared regarding different time periods, including the 1970s, 1980s, 1990s, 2000s, and 2010s. The secondary aims are to identify and study the impact of different risk factors on the development of heart failure after childhood cancer treatment, including the type and dose of cancer therapy, and to examine the potential impact of demographic factors and comorbidities. The participants, Interventions, Comparators, and Outcomes (PICO) framework we used to formulate the research questions is provided below. There are no specific groups of comparison.

- Participants: patients who have survived for at least 5 years after being treated for cancer before the age of 18
- Interventions: childhood cancer treatment
- Comparators: no comparison group
- Outcomes: heart failure incidence and/or prevalence; risk factors for the development of heart failure

### Primary question

What is the incidence and/or prevalence of heart failure in 5-year survivors of childhood cancer posttreatment?

### Secondary question

Can we identify any individual and/or treatment-related risk factors for the development of heart failure in this patient group?

## Methods

This systematic review protocol follows the structure of the recommendation of the Preferred Reporting Items for Systematic Reviews and Meta-Analysis Protocols (PRISMA-P) [26]. The protocol was submitted to the International Prospective Register of Systematic Reviews (PROSPERO) on April 28, 2021, with the registration number CRD42021247622. The reporting of this systematic review will be in accordance with the Meta-Analysis of Observational Studies in Epidemiology (MOOSE) guidelines and in compliance with the Preferred Reporting Items for Systematic Reviews and Meta-Analysis (PRISMA) guidelines [27, 28]. Any amendments to the protocol and rationale during the systematic review will be accounted for in the final report.

### Ethical considerations

The present study will be based solely on previously reported data and will not involve any contact with patients. Hence, there are no concerns that require ethical vetting.

### Eligibility criteria

This systematic review will include population-based studies that reported the incidence and/or prevalence of heart failure after the treatment of any type of childhood cancer. Randomized controlled trials will not be included, since clinical studies of this kind are not expected to address our research questions. Case reports, case series, and other studies not reporting on population-based data will also be excluded since we are interested in the incidence and prevalence of heart failure in populations of CCS compared to the general population. The subjects of interest are patients who received cancer treatment before the age of 18 years and have survived for a minimum of 5 years. The international definition of childhood cancer is a cancer diagnosis for a child aged 0–14 years. However, in many countries in Europe, pediatric health care covers patients until age 18; thus, we decided to include patients treated for cancer before the age of 18. Patients with any cancer type who have received any form of cancer treatment will be included. Animal studies will be excluded from the literature search.

### Study identification/information sources

The following databases will be searched from their inception date to May 17, 2021: MEDLINE, Embase, Scopus, CINAHL, CAB International, AMED, Global health, PsycINFO, Web of Science, and Google Scholar. Grey literature will also be screened to identify studies that were not published in mainstream journals. The reference lists of retrieved papers will be screened to identify possible additional articles of relevance. There will be no language restrictions, and efforts will be made to translate studies reported in languages other than English.

### Search strategy

Our MEDLINE search strategy is provided in Additional file 1, and this will be adapted in searching the other databases.

### Study selection

Retrieved papers will be exported to EndNote, and further screening will be performed using the software program Rayyan. The titles and abstracts of all articles retrieved from the database searches will be screened independently by two reviewers according to the eligibility criteria. After the screening is finished, the reviewers will compare their results, and a third reviewer will arbitrate in case of any disagreements that cannot be resolved by discussion between the two reviewers. Papers that are potentially eligible at this stage will proceed to the next step, which will involve full-text screening, again performed independently by two reviewers, with a third reviewer arbitrating any disagreements. The screening process will be reported in a PRISMA flow diagram and will include the total number of abstracts assessed, as well as the complete number of all full texts retrieved.

### Data extraction and management

A standardized data extraction form for the study will be developed in Excel to retrieve data and applied independently by two reviewers (TB and JB). The extracted information will be based on the PICO structure and include the following:

- Participant characteristics: e.g., sources of subjects, inclusion criteria, and characteristics of the cohort group (age, sex, geographical region, socioeconomic status, ethnicity, comorbidities, ICD cancer diagnosis)
- Exposure to childhood cancer treatment: e.g., anthracyclines, tyrosine kinase inhibitors, alkylating agents, cisplatin, and chest radiation
- Primary outcomes: e.g., heart failure diagnosis (definitions of heart failure from each study will be recorded, and in those with multiple definitions, the proportion of patients will be classified by each method), year of diagnosis, age at diagnosis, incidence or prevalence of heart failure in the cohort, and the frequency of heart failure-related death
- Secondary outcomes: identification of risk factors for the development of heart failure in the population

General information (e.g., reviewer, date of data extraction, record number, author, article title), study characteristics (e.g., study design, study aim), the methods of follow-up, missing data, analyses, and quality assessments will also be collected. The ability of this process to capture the intended data will be piloted with a few studies before a complete data extraction from all included studies is carried out.

When data extraction is completed, the reviewers will compare their results, and a third reviewer (MJ) will arbitrate in cases of any disagreement. If relevant information is missing in a significant article, an effort will be made to contact the authors of the paper so that missing data may be retrieved.

### Quality assessment

The quality and risk of bias in all included studies will be assessed by using the Effective Public Health Practice Project tool (EPHPP) [29]. The EPHPP tool enables the detailed assessment of the strengths and weaknesses of individual studies, as it provides individual ratings for six domains of study quality assessment. For each study, the risk of bias will be categorized as low, moderate, or high. Two independent reviewers will perform the quality appraisal, and a third reviewer will arbitrate in cases of any disagreement.

### Analysis/data synthesis

The descriptive data and characteristics for all included studies will be presented in tables. We will conduct a narrative synthesis of the extracted data and describe the key characteristics and findings of each study. We will search, compare, and contrast the concepts and findings across studies to determine the prevailing concept of the underlying evidence. For studies that are sufficiently homogenous with respect to their methods, populations, designs, interventions/exposures, outcomes, and assessments, we will implement the random-effects meta-analysis using the method of DerSimonian and Laird [30]. We will use the *I*^2^ statistic, which provides an indication of study heterogeneity due to chance in the effect estimates between studies. Furthermore, we will perform subgroup analysis after dividing the study population into groups of age, country of residence, comorbidities, and sex, as well as time period, type of cancer treatment, and years since diagnosis. If a sufficient number of studies is retrieved, we will perform a meta-regression to explore the potential reasons for the heterogeneity in the estimates between studies. We will also undertake a sensitivity analysis to explore any potential scenarios that can change the conclusion of our findings, e.g., by excluding all low-quality studies from the meta-analysis and evaluating whether the results from high-quality studies differ from all studies included together. Finally, the Begg and Egger tests will be used to evaluate the funnel plot asymmetry for the secondary aim of the study in which we evaluate the risk factors for the development of heart failure after childhood cancer treatment [31, 32]. The meta-analysis will be conducted using Stata version 15. All variables will be expressed with a 95% confidence interval (CI).

## Discussion

Heart failure is a late complication after treatment for childhood cancer, which is common, serious, and sometimes fatal. Nevertheless, there are many uncertainties surrounding the epidemiology of this condition. The frequency of heart failure in different subgroups of childhood cancer survivors varies greatly. Although the risk for the development of heart failure increases with the dose of ACs, some patients develop impaired cardiac function even after receiving low doses. This underlines the fact that features other than the cumulative AC dose are important.

In the present review, we will only focus on population-based studies. Thus, we will avoid bias caused by wide variations in the frequency of heart failure due to the differences in specific subpopulations. To our knowledge, there are no previous systematic reviews on heart failure after childhood cancer treatment based on population-based studies. We intend to provide a clearer picture of the epidemiology of heart failure after any childhood cancer treatment. Data on the type and dose of cancer treatment will be collected to study the impact of these factors on the development of heart failure. In addition, we will gather information on demographic features and comorbidities to study to what degree such characteristics may constitute risk factors.

Despite improved survival after treatment for childhood cancer, the incidence of late cardiac complications remains high, especially heart failure. Therefore, it is of great importance to systematically collect data from available studies to generate knowledge about the long-term cardiotoxicity of chemotherapy and radiation that is applied when treating childhood cancer. This systematic review will provide valuable information for future treatment protocols for children with cancer and will also offer guidance for posttreatment cardiac surveillance. Early detection of cardiac abnormalities may allow for early intervention, which is likely to improve the outcomes in this patient population.

## Supplementary Information

**Additional file 1.** MEDLINE search strategies (to be adapted in searching other databases).

**Additional file 2.** PRISMA-P 2015 Checklist.

## Data Availability

Not applicable.

## Abbreviations

ACCF: American College of Cardiology Foundation
AHA: American Heart Association
HF: Heart failure
NYHA: New York Heart Association
ESC: European Society of Cardiology
ACs: Anthracyclines
PROSPERO: International Prospective Register of Systematic Reviews
PRISMA-P: Preferred Reporting Items for Systematic Reviews and Meta-Analysis Protocols
PRISMA: Preferred Reporting Items for Systematic Reviews and Meta-Analysis
MOOSE: Meta-Analysis of Observational Studies in Epidemiology
EPPH: Effective Public Health Practice Project tool
PICO: Participants, Interventions, Comparators, and Outcomes
