# Supplementary material for: Heart failure in childhood cancer survivors—a systematic review protocol
Source: Syst Rev. 2022 Mar 29;11:54. doi: 10.1186/s13643-022-01929-0 (PMC8966343; doi:10.1186/s13643-022-01929-0)
Supplement: Supplementary file 1 — Additional file 1. MEDLINE search strategies (to be adapted in searching other databases). [file 13643_2022_1929_MOESM1_ESM.pdf]

Additional file 1

MEDLINE SEARCH STRATEGIES (TO BE ADAPTED IN SEARCHING OTHER DATABASES)

1. neoplasm.mp. or exp Neoplasms/ or tumor.mp. or tumors.mp. or tumour.mp. or tumours.mp. or leukemia.mp. or exp Leukemia/ or exp Leukemia, Myelomonocytic, Acute/ or exp Leukemia, Myelomonocytic, Juvenile/ or exp Leukemia, B-Cell/ or exp Leukemia, T-Cell/ or Leukemia/ or exp Leukemia, Lymphoid/ or exp Leukemia, Myeloid/ or exp Leukemia, Myeloid, Acute/ or exp Precursor T-Cell Lymphoblastic Leukemia-Lymphoma/ or exp Precursor Cell Lymphoblastic Leukemia-Lymphoma/ or exp Precursor B-Cell Lymphoblastic Leukemia-Lymphoma/ or neoplasia.mp. or neoplasias.mp. or cancer.mp. or cancers.mp. or malignancy.mp. or malignancies.mp. or Malignant Neoplasms.mp. or Malignant Neoplasms.mp.

2. Anthracyclines.mp. or exp Anthracyclines/ or radiation.mp. or exp Radiation/ or radiotherapy.mp. or exp Radiotherapy/ or "radiation therapy".mp. or "antineoplastic agents".mp. or exp Antineoplastic Agents/ or "antineoplastic therapy".mp. or Anthracycline.mp. or exp Anthracyclines/ or aclarubicin.mp. or exp Aclarubicin/ or exp Doxorubicin/ or exp Daunorubicin/ or danorubicin.mp. or carubicin.mp. or exp Carubicin/ or idarubicin.mp. or exp Idarubicin/ or nogalamycin.mp. or exp Nogalamycin/ or plicamycin.mp. or exp Plicamycin/ or "Anticancer Agent".mp. or exp Antineoplastic Agents/ or "Antineoplastic Drugs".mp. or Antineoplastic.mp. or "Antitumor Drug".mp. or "Antitumor Drugs".mp. or "Cancer Chemotherapy Agent".mp. or Antineoplastics.mp. or "Antitumor Agents".mp. or "Cancer Chemotherapy Drugs".mp. or mitoxantrone.mp. or exp Mitoxantrone/ or Acetate.mp. or exp Acetates/ or Hydrochloride.mp. or Novantrone.mp. or exp Antineoplastic Combined Chemotherapy Protocols/ or exp Radiotherapy, Intensity-Modulated/ or exp Radiotherapy, Conformal/ or exp Radiotherapy, Adjuvant/ or exp Radiotherapy Dosage/

3. child.mp. or exp Child/ or children.mp. or pediatric.mp. or exp Pediatrics/ or exp Infant/ or exp Child, Preschool/ or paediatric.mp. or childhood.mp. or exp Adolescent/ or adolescent.mp. or adolescents.mp. or baby.mp. or exp Infant, Newborn/ or infancy.mp. or infanthood.mp. or toddler.mp. or underage.mp. or early life.mp.

4. 1 and 2 and 3

5. "heart failure".mp. or exp Heart Failure/ or "Heart failure diastolic".mp. or exp Heart Failure, Diastolic/ or "Heart failure systolic".mp. or exp Heart Failure, Systolic/ or "heart failure left sided".mp. or "left sided heart failure".mp. or "Heart failure right-sided".mp. or "right sided heart failure".mp. or Cardiomyopathy.mp. or Cardiomyopathies/ or "myocardial failure".mp. or "congestive heart failure".mp. or "heart decompensation".mp. or "cardiac failure".mp. or "left ventricular heart failure".mp. or exp Ventricular Dysfunction, Left/ or exp Ventricular Dysfunction, Right/ or "right ventricular heart failure".mp. or "decompensation heart".mp. or "myocardial disease".mp. or "myocardial disorder".mp. or "myocardial diseases".mp. or "myocardial disorders".mp. or myocardiopathy.mp. or myocardiopathies.mp. or exp Myocardium/ or exp Cardiomyopathies/ or exp Myocytes, Cardiac/ or exp Cardiomyopathy, Hypertrophic/

6. mortality.mp. or exp Mortality/ or death.mp. or exp Death/ or "mortality rate".mp. or "death rate".mp. or "mortality rates".mp. or "death rates".mp. or "excess mortality".mp. or "fatality rate".mp. or incidence.mp. or exp Incidence/ or "incidence rate".mp. or "incidence rates".mp. or exp Prevalence/ or prevalence.mp. or "prevalence rate".mp. or "prevalence rates".mp. or "incidence proportion".mp. or "point prevalence".mp or "period prevalence".mp or "life-time prevalence".mp or "cumulative incidence".mp or Long-Term Cancer Survivors.mp. or exp Cancer Survivors/ or Survivors, Cancer.mp. or Cancer Survivor, Long-Term.mp. or Cancer Survivors, Long-Term.mp. or Long Term Cancer Survivors.mp. or Long-Term Cancer Survivor.mp. or Survivor, Long-Term Cancer.mp. or Survivors, Long-Term Cancer.mp. or Survivors of Childhood Cancer.mp. or Cancer Survivor, Childhood.mp. or Cancer Survivors, Childhood.mp. or Childhood Cancer Survivor.mp. or Childhood Cancer Survivors.mp.

7. 5 and 6

8. 4 and 7
